# Psychosocial correlates of risky sexual behaviour amongst students in Niger Delta University, Bayelsa

**DOI:** 10.11604/pamj.2021.38.7.27312

**Published:** 2021-01-05

**Authors:** Ikenna Desmond Ebuenyi, Uzoechi Eze Chikezie, Eunice Anyalewechi Nwoke

**Affiliations:** 1 Assisting Living & Learning Institute, Department of Psychology, Maynooth University, Maynooth, Ireland,; 2 Department of Mental Health, Faculty of Clinical Sciences, Niger Delta University, Amassoma Bayelsa State, Nigeria,; 3 Department of Public Health Technology, School of Health Technology, Federal University of Technology, Owerri Imo State, Nigeria

**Keywords:** Risky sexual behaviour, depression, psychosocial correlates, Niger Delta

## Abstract

**Introduction:**

globally young people constitute one quarter of the population. They are the most vibrant and productive sector, but they are also prone to more risky sexual behaviour (RSB) with attendant negative consequences. In the Niger Delta Region of Nigeria, persistent conflicts and socioeconomic difficulty predisposes young people to risky conducts including RSB. The aim of this study is to explore the psychosocial correlates of risky sexual behaviour amongst students in the Niger Delta University, Bayelsa.

**Methods:**

we undertook a descriptive cross-sectional survey of students in the university. A multistage random sampling technique was used to recruit 400 students who completed a self-administered questionnaire. The data collected was analyzed using SPSS version 20.0.

**Results:**

out of the 400 students, 64.3% (257) engaged in RSB and 46.8% (187) were currently engaging in RSB. Rates of self-reported depression, suicidal ideation and attempt were 62.3% (249), 18.0% (72) and 14.3% (54) respectively. Independent correlates of RSB include being older than 19 years (aOR. 2.82; 95% C.I. 1.44 -5.51), male gender (aOR. 1.70; 95% C.I. 1.08-2.66), having depression (aOR. 1.83; 95% C.I. 1.15-2.92), being diagnosed with a sexually transmitted disease (STD)/HIV (aOR. 2.08; 95% C.I. 1.12-3.86), and having been taught about condoms (aOR. 1.80; 95% C.I. 1.13-2.86).

**Conclusion:**

risky sexual behaviours have negative psychosocial and health implications. Regular and continuous health education targeted at young people is essential to reduce the social and health effects associated with RSB.

## Introduction

Young people between the ages of 10-24 represent about a quarter of the world´s population and globally they engage in risky sexual behaviour (RSB) with diverse psychosocial and health consequences [1, 2]. Psychological conditions such as depression, low self-esteem and suicidality and social effects such as unwanted pregnancy and drop out of school are associated with RSB in young people [1-3]. Risky sexual behaviours such as unprotected sexual intercourse, multiple sexual partners and sexual intercourse under the influence of alcohol or drugs have psychosocial and health implications [2]. Risky sexual behaviours with persons with sexually transmitted infections/disease (STI/STD) such as Human immunodeficiency virus (HIV) predisposes to infectious diseases [3]. Studies have identified an association between RSB and psychiatric disorders in young people [3-7]. Depression, low self-esteem and suicidality are reported consequences of RSB in young people [4, 5, 8, 9] In addition ,young people who engage in RSB are more likely to drop out of school or have unwanted pregnancies [10, 11].

Studies amongst young people in Nigerian universities shows marked levels of RSB. Prevalence of RSB ranged from 55% to 85% [12-14]. Young people in the Niger Delta Region (NDR) of Nigeria engage in unhealthy sexual behaviour characterized by early age at sexual initiation, unsafe sex and multiple sexual partners [15-17]. The local socio-economic condition exerts extra pressure on the adolescent with negative reproductive health consequences. This high level of unsafe sexual behaviour has led to a high rates of STI, unintended and unwanted pregnancies, illegal abortions and impaired academic performance [12-16].

Studies on RSB in Nigerian youths abound [12-14, 16] but few have explored the psychosocial correlates of such behaviours. Psychosocial correlates and predictors of RSB are of public health relevance to society and health care providers [1, 2]. Hence, this study aimed to identify the psychosocial correlates of risky sexual behaviour amongst students in Niger Delta University (NDU), Bayelsa State. It will provide essential information for future longitudinal studies to identify the exact mechanism of the associations as well help in the reduction of public health and socio-economic burden on the region and the nation in general.

## Methods

**Study design and site:** this was a cross sectional study conducted in Niger Delta University, Bayelsa State. The NDU was established in 2000 and is a Bayelsa State Government funded university which is located in Wilberforce Island (an island of great historic significance) about 30 kilometers from Yenagoa - Bayelsa State capital [18]. NDU is the largest tertiary institution in the state with a population of about 20,000 students and 3518 academic and non-academic staff [18].

**Sample size and selection:** the study population were young people (male and female) between the ages of 15 to 24 years in Niger Delta University Bayelsa. The total student population is about 20,000. A total of 400 randomly sampled youths who gave consent were selected for the study using the Yarmane sample size table for large populations [19]. A multistage sampling method was used to recruit the study participants.

**Data collection:** a researcher designed questionnaire was used for data collection and were self-administered with the assistance of trained field assistants after being pre-tested. The questionnaire covered the following areas: demographic data, sexual behaviour, risky sexual behaviour (for example use of drugs or alcohol prior to sexual intercourse, multiple sexual partners and contraception usage), psychosocial factors and mental health status. Sexual attitudes, self-esteem, depression, suicidal ideation and attempts were assessed via self-report. The measures were adapted for use in the study setting as used in a previous study [9]. Engaging in RSB was determined using the following criteria: Ever engaged in RSB condition is: Ever had sex and (sex for money or not using condoms or multiple partners or sex under influence of alcohol) [2]. Currently engaging in RSB condition is: engaged in sex in the last one month and (sex for money or not using condoms or multiple partners or sex under influence of alcohol).

**Statistical analysis:** all the analysis was conducted using SPSS version 20.0. The relationship between each of the explanatory (Socio-demographic and psycho-social variables) and outcome variable (engaging in RSB) were examined using cross tabulation reporting chi-square and P-value to investigate an initial “unadjusted” association. Multivariable analysis was performed using logistic regression model by spontaneous selection of variables found to be significantly associated (P-value <0•2) with engaging in RSB from bivariate analysis. The level of statistical significance was at p<0.05.

**Ethical considerations:** ethical approval for the study was obtained from the research and ethics committee of the Niger Delta University (NDUTH/REC/005/2015). Informed consent was sought and obtained from all study participants.

## Results

**Socio-demographic characteristics of study participants:** Table 1 presents the socio-demographic characteristics of the study participants disaggregated by RSB. A total 400 participants completed the survey with a response rate of 94%. More than half (59.3%) were females and majority (83.5%) of study participants were aged 19 years and above. In terms of marital status, 85.3% (341) were single, 8.8% (35) were married and 6.0% (24) were either separated or divorced. Majority (81.0%) were undergraduate students. Rates of self-reported depression, suicidal ideation and attempt 62.3% (249), 18.0% (72) and 14.3% (54) respectively. Majority (45.6%) rated their self-esteem as moderate while 39.0% (147) and 15.4% (58) rate their self-esteem high and low respectively. Only 22.5% (90) of the students have ever dropped out of school. About 18.0% (72) of study participants had been diagnosed of STD/HIV and 46.8% (187) indicated that they have been taught about condoms in school.

**Table 1:** socio-demographic characteristics of study participants

Variable	Category	Engaged in Risky Sexual Behaviour		Total Population	p-value
		No	Yes		
Age	≤18 years	50(75.8%)	16(24.2%)	66(16.5%)	P<0.001
	≥19 Years	163(48.8%)	171(51.2%)	334(83.5%)	
Gender	Female	143(60.3%)	94(39.7%)	237(59.3%)	P=0.001
	Male	70(42.9%)	93(57.1%)	163(40.8%)	
Marital status	Single	186(54.5%)	155(45.5%)	341(85.3%)	P=0.400
	Married	17(48.6%)	18(51.4%)	35(8.8%)	
	Separated/Divorced	10(41.7%)	14(58.3%)	24(6.0%)	
Level of education	Undergraduate	186(57.4%)	138(42.6%)	324(81.0%)	P=0.001
	Postgraduate	27(35.5%)	49(64.5%)	76(19.0%)	
Religion	Christian	187(53.7%)	161(46.3%)	348(87.0%)	P=0.001
	Islam	21(75.0%)	7(25.0%)	28(7.0%)	
	Africa Traditional Religion	4(25.0%)	12(75.0%)	16(4.0%)	
	Other	1(12.5%)	7(87.5%)	8(2.0%)	
Suicidal Ideation	Yes	35(48.6%)	37(51.4%)	72(18.0%)	P=0.384
	No	178(54.3%)	150(45.7%)	328(82.0%)	
Suicide attempt	No	180(52.5%)	163(47.5%)	343(85.8%)	P=0.448
	Yes	33(57.9%)	24(42.1%)	57(14.3%)	
Depression	Yes	123(49.4%)	126(50.6%)	249(62.3%)	P=0.047
	No	90(59.6%)	61(40.4%)	151(37.8%)	
Level of self-esteem	High	77(52.4%)	70(47.6%)	147(39.0%)	P=0.925
	Low	32(55.2%)	26(44.8%)	58(15.4%)	
	Moderate	90(52.3%)	82(47.7%)	172(45.6%)	
Dropped out of school	Yes	49(54.4%)	41(45.6%)	90(22.5%)	P=0.796
	No	164(52.9%)	146(47.1%)	310(77.5%)	
Diagnosed with HIV/STD	No	187(57.0%)	141(43.0%)	328(82.0%)	P=0.001
	Yes	26(36.1%)	46(63.9%)	72(18.0%)	
Friends influence decision	No	161(55.9%)	127(44.1%)	288(72.0%)	P=0.088
	Yes	52(46.4%)	60(53.6%)	112(28.0%)	
Talk important things with parents	No	69(48.6%)	73(51.4%)	142(35.5%)	P=0.166
	Yes	144(55.8%)	114(44.2%)	258(64.5%)	
Taught about HIV/AIDS in school	No	54(54.5%)	45(45.5%)	99(24.8%)	P=0.766
	Yes	159(52.8%)	142(47.2%)	301(75.3%)	
Taught about condoms in school	No	124(58.2%)	89(41.8%)	213(53.3%)	P=0.034
	Yes	89(47.6%)	98(52.4%)	187(46.8%)	

**Patters of risky sexual behaviour:** Table 2 presents the patterns of RSB. Among the respondents 78% (312) had ever engaged in sex, 55.5% (222) had engaged in sex in the past one month. Nearly one third (32.7%) had engaged in sex with one partner, while 12.5% (39) had engaged with six or more partners. Over a third (38.5%) indicated that they have engaged in sex with multiple partners, 31.4% (98) indicated that they had sex under influence of alcohol, while 22.4% (70) said they had sex for money.

**Table 2:** patterns of risky sexual behaviours

Variable	Category	Frequency	Percentage (%)
Ever Had Sex (N=400)	No	88	22.0
	Yes	312	78.0
Sex in the past one Month (N=400)	No	178	44.5
	Yes	222	55.5
Number of Partners in the past one month (N=312)	None	90	28.8
	One	102	32.7
	Two	37	11.9
	Three	27	8.7
	Four	16	5.1
	Five	1	0.3
	6 or More	39	12.5
Engage in Sex with Multiple partners (N=312)	No	192	61.5
	Yes	120	38.5
Condom used during last sexual encounter (N=312)	No	190	60.9
	Yes	122	39.1
Sex Under Influence of alcohol (N=312)	No	214	68.6
	Yes	98	31.4
Sex for Money (N=312)	No	242	77.6
	Yes	70	22.4

**Prevalence of risky sexual behaviour:** Table 3 presents the prevalence of risky sexual behaviour. More than half (64.3% 95% C.I. 59.5-69.0) had ever engaged in risky sexual behaviour, while 46.8% (95% C.I. 42.0-51.5) were currently engaging in risky sexual behaviour.

**Table 3:** prevalence of risky sexual behaviour

Risky Sexual Behaviour	Frequency (N=400)	Percentage (%)	95% C.I.	
			Lower	Upper
Ever engaged in Risky Sexual Behaviour	257	64.3	59.5	69.0
Currently engaging in Risky sexual Behaviour	187	46.8	42.0	51.5

**Correlates of risky sexual behaviours:** Table 4 presents the results of correlates of risky sexual behaviour both crude/ before (bivariate analysis)) and adjusted (multivariable analysis). At the bivariate level, participants who were older (≥ 19), male gender, having depression, being diagnosed of STD/HIV, having being taught about condoms, postgraduate respondents, and not agreeing that abstinence from alcohol can reduce RSB were significantly associated with RSB (P < 0.05). Participants whose religion was Islam were less likely to engage in RSB as compared to those from other religions. After adjusting for all variables that were significantly associated with RSB at bivariate level (p < 0.2). The risk of engaging in RSB was about three times more (adjusted odds ratio [aOR]. 2.82; 95% confidence interval [C.I.] 1.44-5.51) among those aged ≥19 years as compared to those who were aged 18 years and below. Males were 1.77 times more likely to engage in RSB (aOR. 1.70; 95% C.I. 1.08-2.66) as compared to females. Having depression, being diagnosed with STD/HIV, being taught about condoms in school was significantly associated with engaging in RSB (p < 0.05). Participants who felt that abstinence from alcohol cannot reduce RSB were more likely to engage in RSB. Participants belonging to other religion, traditional African and Christians were more likely to engage in RSB as compared to those from Islam.

**Table 4:** correlates of risky sexual behaviours

Parameter	Category	cOR(95% C.I.)	p-value	aOR(95% C.I.)	p-value
Age	≥19 Years	3.28(1.79-5.99)	**<0.001**	2.82(1.44-5.51)	**0.002**
	≤18 years (Ref)	1		1	
Gender	Male	2.02(1.35-3.03)	**0.001**	1.70(1.08-2.66)	**0.021**
	Female (Ref)	1		1	
Marital status	Separated/Divorced	1.68(0.73-3.89)	0.226	n/s	n/s
	Married	1.27(0.63-2.55)	0.500	n/s	n/s
	Single (Ref)	1		1	
Level of education	Postgraduate	2.45(1.46-4.11)	**0.001**	2.06(1.12-3.81)	0.020
	Undergraduate (Ref)	1		1	
Religion	Other	21.00(2.18-201.88)	**0.008**	23.82(2.03-279.16)	**0.012**
	Traditional	9.00(2.18-37.18)	**0.002**	6.56(1.41-30.55)	**0.017**
	Christian	2.58(1.07-6.23)	**0.035**	2.99(1.15-7.81)	**0.025**
	Islam (Ref)	1		1	
Suicide Ideation	No	0.80(0.48-1.33)	0.384	n/s	n/s
	Yes (Ref)	1		1	
Suicide Attempts	Yes	0.80(0.46-1.42)	0.448	n/s	n/s
	No (Ref)	1		1	
Depression	Yes	1.51(1.00-2.28)	**0.048**	1.83(1.15-2.92)	**0.011**
	No (Ref)	1		1	
Level of self-esteem	Moderate	1.00(0.64-1.56)	0.992	n/s	n/s
	Low	0.89(0.49-1.65)	0.718	n/s	n/s
	High (Ref)	1		1	
Dropped out of school	No	1.06(0.66-1.70)	0.796	n/s	n/s
	Yes (Ref)	1		1	
Diagnosed with HIV/STD	Yes	2.35(1.38-3.98)	**0.002**	2.08(1.12-3.86)	**0.020**
	No (Ref)	1		1	
Friends influence decision	Yes	1.46(0.94-2.27)	0.089	1.47(0.89-2.42)	0.131
	No (Ref)	1		1	
Talk important things with parents	Yes	0.75(0.50-1.13)	0.166	0.66(0.40-1.08)	0.096
	No (Ref)	1		1	
Taught about HIV/AIDS in school	Yes	1.07(0.68-1.69)	0.766	n/s	n/s
	No (Ref)	1		1	
Taught about condoms in school	Yes	1.53(1.03-2.28)	**0.034**	1.80(1.13-2.86)	**0.014**
	No (Ref)	1		1	
Alcohol Abstinence can reduce RSB	No	2.02(1.35-3.03)	**0.001**	2.25(1.40-3.61)	**0.001**
	Yes (Ref)	1		1	
Abstinence can reduce risky sexual behavior	Yes	0.63(0.41-0.98)	**0.043**	1.05(0.62-1.76)	0.856
	No (Ref)	1		1	

Note: cOR-Crude Odds Ratio; aOR.-Adjusted Odds Ratio; Ref:-Reference Category; n/s-Not significant

## Discussion

This study conducted in a tertiary institution in the Niger Delta Region identified some psychosocial correlates of RSB amongst young people in Niger Delta University Bayelsa. This study recorded a 64.3% prevalence of RSB which is higher than the 13.8%-39.8% reported in the United States of America (USA) [20]. However, it is less than the 85% in Ile-Ife, South west Nigeria [21]. These differences in prevalence may be on account of the study setting, design and population specific factors.

In our study, depression was noted as an independent correlate of RSB. The relationship between RSB and psychological problems are well documented; however, the direction of the relationship is contentious. While some studies aver that psychological problems predict RSB [6, 22, 23], other studies have recorded psychological problems as consequences of RSB [3, 5, 7-9, 24]. The finding from our study is corroborated by a large longitudinal study by Hallfors and colleagues who recorded RSB as a predictor of depression especially in young girls [7]. According to Malhotra, young people who engage in RSB are more likely to suffer from depression [3]. The World Health Organization [2] reports that depression is the most prevalent neurological condition amongst young people who engage in RSB. Studies suggest that RSB often predispose young people to pain and suffering, sense of betrayal and abandonment and consequently depression [3, 10].

Suicide has been identified as a sequel to depression [2]. Although this study did not record a significant relationship between RSB and suicidal ideation or attempt, it found that self-reported suicidal ideation was slightly higher in those who engage in RSB. Studies suggest that suicidality is heightened in young people with RSB [8, 25]. It is pertinent to note that although in this study, low self-esteem was not found to be significantly associated with RSB, rates of self-esteem were lower amongst study participants who engage in RSB. Similarly, Searle [9] reported higher self-esteem amongst female who do not engage in RSB.

Several studies have documented social and health consequences of RSB. In this study, being diagnosed of STD/HIV was found to be an independent correlate of RSB. STD and or HIV are known consequences of RSB and the finding from this study is corroborated by previous studies on the subject [26, 27]. This showed that being 19 years and above and being male correlated with RSB. A study on unprotected sexual intercourse amongst HIV patients in the Niger Delta showed that younger age and female gender were predictors of RSB. However, the findings from our study are similar to the higher rates of RSB amongst males reported by Ishida and colleagues [28]. This study also noted that having been taught about condom correlated with RSB. Although knowledge and use of condom is protective against unprotected sexual intercourse [26], it may not be helpful for other markers of RSB such as multiple sexual partners, sex under the influence of alcohol and sex for money. Also, in this study, 39.1% of study participants used a condom in their last sexual encounter. Although this is comparable and higher than the 32% recorded in Rivers, Nigeria [14], it is less than the 60.2% reported in the USA [20]. The health implications of these low rates of condom use in our region are worrisome.

Finally, we found that study participants that believed that alcohol cannot reduce RSB were more likely to engage in it. Previous studies have reported the relationship between RSB and alcohol [2, 14]. Also, we noted that participants who self-reported Islam as their religion were less likely to engage in risky sexual behaviour compared to others from other religion. The role of religiosity on risky sexual and health behaviours though a contentious topic is not new [28-30]. A study among Iranian university students found that religiosity was associated with less risky sexual behaviours [29] while another study of adolescents in USA indicated that self-reported religiosity predicted less risky health behaviours [30].

In spite of the findings of our study, it has some limitations. The cross-sectional nature of the study and the reliance on self-report for sexual behaviour and psychological factors are major limitations. The cross-sectional nature of the study limits the observations to mere associations. Hence, the psychosocial consequences may not be attributed to RSB alone. Several other factors may be at play and it may require a longitudinal study to determine causality or temporality between RSB and the psychosocial factors enumerated in the study.

## Conclusion

This study has demonstrated high rates of self-reported RSB among young people in Niger Delta University. Depression was identified as an independent correlate of RSB. Further studies are essential to determine the relationship between RSB and psychological manifestations. There is need to intensify local and national levels efforts to provide young people with sexual and reproductive health education to stem the tide of RSB and the attendant psychosocial and health implications.

### What is known about this topic

- Globally and in the Niger Delta Region of Nigeria, young persons engage in risky sexual behaviours;
- Risky sexual behaviours have health and psychosocial consequences.

### What this study adds

- Depression, male gender are independent correlates of risky sexual behaviour amongst university students in the Niger Delta Region of Nigeria;
- A previous diagnosis of a sexually transmitted infection and having been taught about the use of condoms is associated with an increase in risky sexual behaviour;
- The heightened rates of risky sexual behaviours amongst young persons in the Niger Delta Region of Nigeria require urgent public health interventions.
